# AI-integrated Screening to Replace Double Reading of Mammograms: A Population-wide Accuracy and Feasibility Study

**DOI:** 10.1148/ryai.230529

**Published:** 2024-09-04

**Authors:** Mohammad T. Elhakim, Sarah W. Stougaard, Ole Graumann, Mads Nielsen, Oke Gerke, Lisbet B. Larsen, Benjamin S. B. Rasmussen

**Affiliations:** From the Department of Radiology (M.T.E., L.B.L., B.S.B.R.), Department of Nuclear Medicine (O. Gerke), and CAI-X–Centre for Clinical Artificial Intelligence (B.S.B.R.), Odense University Hospital, Kløvervænget 10, Entrance 112, 2nd Floor, 5000 Odense C, Denmark; Department of Clinical Research, Research and Innovation Unit of Radiology, University of Southern Denmark, Odense, Denmark (M.T.E., S.W.S., O. Graumann, O. Gerke, B.S.B.R.); Department of Radiology, Aarhus University Hospital, Aarhus, Denmark (O. Graumann); Department of Clinical Research, Aarhus University, Aarhus, Denmark (O. Graumann); and Department of Computer Science, University of Copenhagen, Copenhagen, Denmark (M.N.).

**Keywords:** Mammography, Breast, Neoplasms-Primary, Screening, Epidemiology, Diagnosis, Convolutional Neural Network (CNN)

## Abstract

Mammography screening supported by deep learning–based artificial intelligence (AI) solutions can potentially reduce workload without compromising breast cancer detection accuracy, but the site of deployment in the workflow might be crucial. This retrospective study compared three simulated AI-integrated screening scenarios with standard double reading with arbitration in a sample of 249 402 mammograms from a representative screening population. A commercial AI system replaced the first reader (scenario 1: integrated AI_first_), the second reader (scenario 2: integrated AI_second_), or both readers for triaging of low- and high-risk cases (scenario 3: integrated AI_triage_). AI threshold values were chosen based partly on previous validation and setting the screen-read volume reduction at approximately 50% across scenarios. Detection accuracy measures were calculated. Compared with standard double reading, integrated AI_first_ showed no evidence of a difference in accuracy metrics except for a higher arbitration rate (+0.99%, *P* < .001). Integrated AI_second_ had lower sensitivity (−1.58%, *P* < .001), negative predictive value (NPV) (−0.01%, *P* < .001), and recall rate (−0.06%, *P* = .04) but a higher positive predictive value (PPV) (+0.03%, *P* < .001) and arbitration rate (+1.22%, *P* < .001). Integrated AI_triage_ achieved higher sensitivity (+1.33%, *P* < .001), PPV (+0.36%, *P* = .03), and NPV (+0.01%, *P* < .001) but lower arbitration rate (−0.88%, *P* < .001). Replacing one or both readers with AI seems feasible; however, the site of application in the workflow can have clinically relevant effects on accuracy and workload.

**Keywords:** Mammography, Breast, Neoplasms-Primary, Screening, Epidemiology, Diagnosis, Convolutional Neural Network (CNN)

*Supplemental material is available for this article.*

Published under a CC BY 4.0 license.

See also commentary by Suri in this issue.

**Summary**

Evaluation of three scenarios of simulated artificial intelligence (AI)–integrated screening in an entire mammography screening population showed that replacing one reader or both readers with AI in a double-reading setting can reduce workload without reducing cancer detection accuracy.

**Key Points**

■ Accuracy and feasibility of three simulated scenarios of artificial intelligence (AI)–integrated screening were evaluated in a large-scale cohort from population-wide double-read mammography screening.
■ Replacing both readers for triaging of low- and high-risk cases or replacing the first reader fully with AI reduced the volume of screening reads by 49.7% and 48.8%, respectively, without reducing cancer detection accuracy.
■ Fully replacing the second reader with AI reduced the volume of screening reads by 48.7% and the number of recalls by 2.2%, but at a cost of a reduced sensitivity (−1.5%, *P* < .001).

## Introduction

A growing body of evidence substantiates promising accuracy levels of stand-alone artificial intelligence (AI) solutions for breast cancer detection, comparable to or exceeding that of radiologists within mammography screening (1). However, the generalizability of AI performance to real-life screening has been questioned. This is a result of methodologic limitations and high risk of bias in the literature, mainly due to small, cancer-enriched or nonrepresentative study samples, inadequate reference standards, and lack of comparisons with screen readers in real-world practice (2–4). A recent multicenter study performed on a consecutive cohort representative of a screening population showed equivalent accuracy between a commercial AI solution as a stand-alone AI and first readers when the AI score threshold was matched at mean first reader specificity (AI_spec_) (5). Replacing one human reader in the context of serial, blinded double reading with AI is considered among radiologists as an optimal deployment site within screening (6), but little work has been done to assess the accuracy and feasibility of such AI-integrated screening scenarios in representative real-life screening populations. To explore optimal deployment sites of AI in the screening workflow, this study aimed to evaluate the accuracy and feasibility of three different AI-integrated screening scenarios replacing one or both radiologists compared with standard double reading with arbitration in an entire screening population.

## Materials and Methods

This retrospective study was approved by the national ethics committee (identifier D1763009). The need for individual informed consent was waived. The study is reported in accordance with the Checklist for Artificial Intelligence in Medical Imaging (ie, CLAIM) guidelines (7). Further details on methodologic aspects, other than those described in the following sections, are provided in Appendix S1.

### Study Design and Sample

The study was designed as a multicenter study of detection accuracy and feasibility on a retrospective cohort including all screening mammograms in the Region of Southern Denmark between August 4, 2014, and August 15, 2018, as previously reported (5). In brief, a study population of 272008 consecutive screening mammograms was retrieved, including original binary assessments of normal or abnormal (recall) by breast radiologists through independent, blinded double reading with arbitration for disagreements. Exclusion criteria were insufficient image quality, missing images, lack of follow-up, or image data types not supported by the AI.

### Reference Standard

Follow-up information was acquired by matching with two validated national breast cancer and screening databases (8,9) accessed through the Danish Clinical Quality Program–National Clinical Registries (ie, RKKP). Positive and negative cancer outcomes were determined through histopathology-verified breast cancer or cancer-free follow-up, respectively, up until the next consecutive screening or within 24 months.

### AI System

The Conformité Européenne–marked and U.S. Food and Drug Administration–approved deep learning–based AI system Lunit INSIGHT MMG version 1.1.7.1, intended for concurrent reading aid for breast cancer detection at mammography, processed all study cases. The AI system gave a lesion score indicating the likelihood of presence of malignancy in specific regions in each standard view. The highest lesion score from the available views yielded a per-breast level abnormality score from 0% to 100%, which was used as a representative examination-level score representing the maximum score in either breast of each woman. This examination-level score was dichotomized into an AI score with thresholds selected for each scenario (as further elaborated below) to enable comparability with human reader outcomes.

### AI-integrated Screening Scenarios

Three different AI-integrated screening scenarios were explored and compared with the standard radiologist double reading with arbitration (ie, combined reading) (Figure). To ensure better comparability of the scenarios in terms of accuracy and feasibility, the intervention and thresholds for the AI system were chosen partly based on maintaining a workload reduction in the double reading process of about 50% in each scenario. In scenario 1 (integrated AI_first_), the first reader was replaced by the AI system with a validated threshold matched at mean first reader specificity from the study sample (AI_spec_), corresponding to an AI score of 80.99%. Outcomes above the threshold were considered as recalls, as previously detailed (5). As for cases of disagreement between the AI and second reader for which original arbitrations were absent, the arbitration decision was simulated to match the same accuracy level of the original arbitrator to maintain realistic estimates of the reading outcomes. In both scenarios 1 and 2, available original arbitrations were disregarded in cases where AI agreed with the reader. In scenario 2 (integrated AI_second_), the AI system replaced the second reader with the same threshold and approach to arbitrations as described for scenario 1. In cases of disagreements between the AI and first reader for which original arbitrations were absent, simulated arbitrations were applied in the same approach as described for scenario 1. In scenario 3 (integrated AI_triage_), AI was applied as a stand-alone tool to triage screenings as low risk, moderate risk, and high risk. The AI system replaced both readers in the assessment of low-risk and high-risk screenings that were sent to no recall and recall, respectively, while the original combined reading was applied for moderate-risk cases. The combination of high-risk and low-risk thresholds that ensured 50% of screenings being placed in the moderate-risk group was chosen by fixing an equal recall rate to that of the combined reading to avoid increasing the workload. This yielded high-risk and low-risk thresholds at an AI score of 95.29% or less and less than 3.36%, respectively.

**Figure 1:**
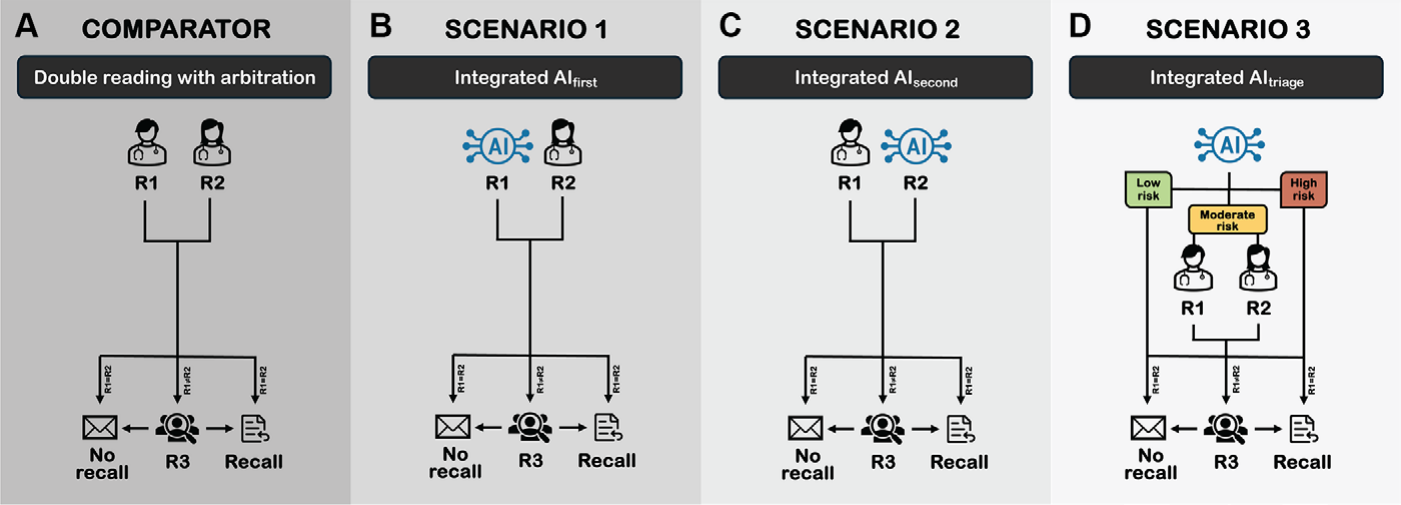
Schematic illustration of the standard screening workflow and the study scenarios. **(A)** Standard blinded double reading by the first reader (R1) and the second reader (R2) with arbitration (R3), that is, combined reading. **(B)** In scenario 1, integrated AI_first_, the artificial intelligence (AI) system was simulated to replace the first reader. In cases of disagreement between the AI and the second reader, for which no original arbitration was available, the arbitration decision was simulated at an accuracy level that approximated the original arbitrator’s accuracy from the study sample. **(C)** In scenario 2, integrated AI_second_, the AI system was simulated to replace the second reader. Cases of disagreements between the AI and the first reader, in cases with absent original arbitrations, were resolved as described for scenario 1. **(D)** In scenario 3, integrated AI_triage_, the AI system was simulated to be a stand-alone reading tool that triaged screenings into low, moderate, and high risk for cancer. In low- and high-risk cases, the decision of the AI was final without any other reading, leading to no recall and recall, respectively. In moderate-risk cases, the decision of the original combined reading including arbitration was used as the outcome.

### Performance Metrics and Statistical Analysis

The metrics of accuracy were sensitivity and specificity as coprimary end points and positive predictive value (PPV), negative predictive value (NPV), recall rate, and arbitration rate as secondary end points, supplemented by the areas under the receiver operating characteristic curves (AUCs). The cancer detection rate (CDR) was calculated as the number of true-positive detected cancers per 1000 screening mammograms. Binomial proportions for accuracy of the combined reading and each AI-integrated screening scenario were calculated and supplemented by 95% Clopper-Pearson (“exact”) CIs. McNemar test and a score test for the comparison of predictive values in paired designs (10) were used to compare the accuracy of the combined reading and the AI-integrated screening scenarios. The *t* test was used to evaluate associations with continuous variables (age). A *P* value less than .00833 was considered statistically significant in the analysis of the coprimary end points to adjust for multiple testing across the three scenarios, while a *P* value less than .05 was considered statistically significant for the other accuracy metrics. Stata/SE (release 17; StataCorp) was used for all statistical analyses.

## Results

### Sample Characteristics

The final study sample consisted of 249 402 screenings in 149 495 female individuals with a mean age of 59.3 years ± 5.9 (SD). There were 2033 cancers, with 1475 (72.6%) screen-detected cancers and 558 (27.4%) interval cancers, as detailed previously (5). Overall, 22 606 mammograms (8.31%) were excluded from the original study population (*n* = 272 008) because of technical recall (*n* = 9), no match in clinical databases (*n* = 4734), lack of follow-up (*n* = 1960), irretrievable images (*n* = 1969), unsupported image type for the AI (*n* = 13 923), and rejection by the AI system (*n* = 11) (5). A detailed study flowchart is shown in Figure S1.

### Performance Evaluation of AI-integrated Screening

Table 1 shows the cancer detection accuracy of combined reading (comparator) and the AI-integrated screening scenarios, as well as that of first reader and second reader to provide context. Cross tabulation of the screen reading outcomes in the three scenarios by the reference standard results in comparison to the combined reading is presented in Table 2. Receiver operating characteristic curves and corresponding AUC values for all scenarios are shown in Figure S2. Compared with combined reading, integrated AI_first_ in scenario 1 had a higher CDR (6.09 vs 6.03) but showed no evidence of a difference for all outcome measures except for a higher arbitration rate (+0.99%, *P* < .001). In scenario 2, integrated AI_second_ had a lower CDR (5.91 vs 6.03), sensitivity (−1.58%, *P* < .001), NPV (−0.01%, *P* < .001), and recall rate (−0.06%, *P* = .04) but a higher PPV (+0.03%, *P* < .001) and arbitration rate (+1.22%, *P* < .001). In scenario 3, integrated AI_triage_ showed higher CDR (6.14 vs 6.03), sensitivity (+1.33%, *P* < .001), PPV (+0.36%, *P* = .03), and NPV (+0.01%, *P* < .001) but a lower arbitration rate (−0.88%, *P* < .001). The reduction in human screen reads in scenarios 1, 2, and 3 was −48.8%, −48.7%, and −49.7%, respectively (Table 2). Analysis of cancer subgroups showed significantly lower detection accuracy for screen-detected cancers and higher detection accuracy for interval cancers in all three scenarios (Table 3). The distribution across risk groups in scenario 3 is shown in Table S1.

**Table 1:**
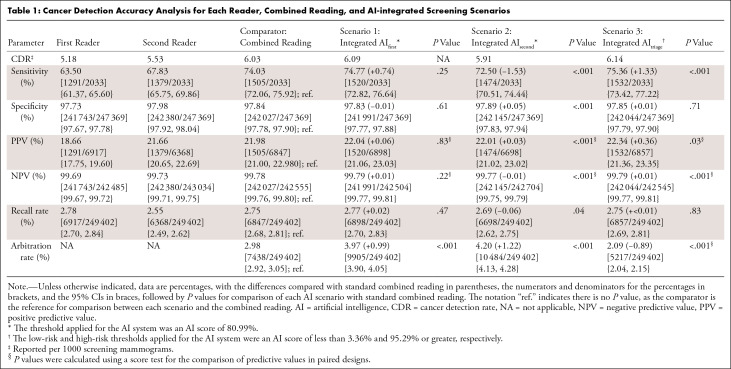
Cancer Detection Accuracy Analysis for Each Reader, Combined Reading, and AI-integrated Screening Scenarios

**Table 2:**
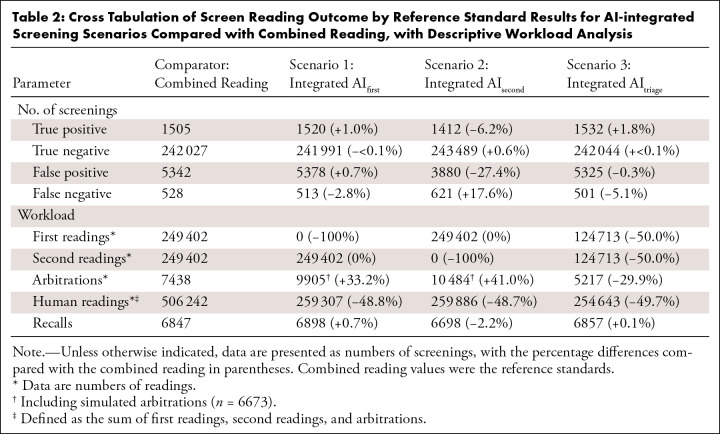
Cross Tabulation of Screen Reading Outcome by Reference Standard Results for AI-integrated Screening Scenarios Compared with Combined Reading, with Descriptive Workload Analysis

**Table 3:**
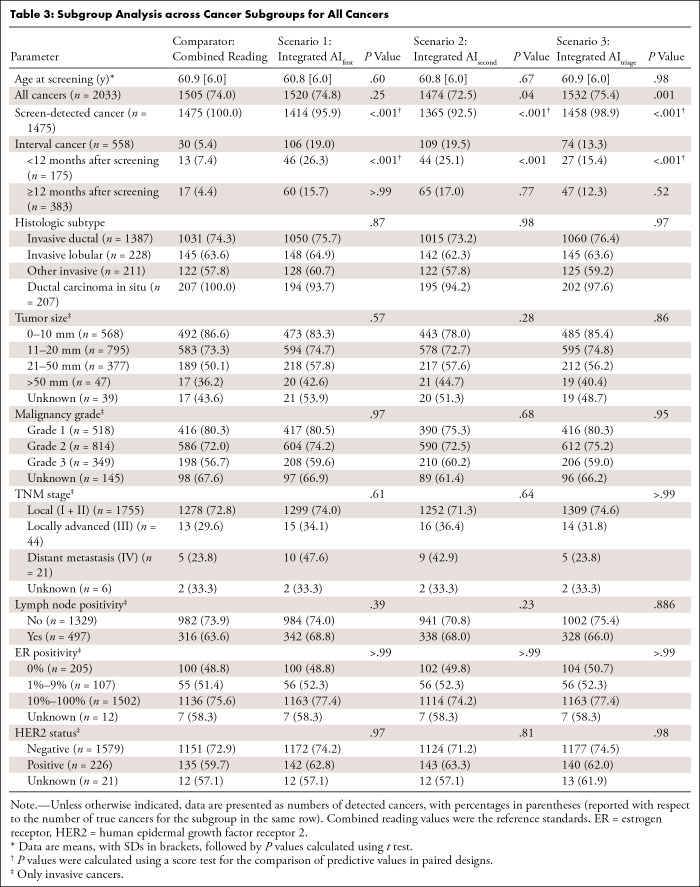
Subgroup Analysis across Cancer Subgroups for All Cancers

## Discussion

AI-integrated mammography screening was feasible across all scenarios in terms of workload reduction, with an approximately 50% decrease in screen-reading volume, including arbitrations and stable or reduced recall rates. Given that second readers were more experienced in the current study population (5) and had higher detection performance across all accuracy measures than the first reader, a lower CDR was expected in scenario 2 for integrated AI_second_, especially because the selected AI threshold was the same as in scenario 1. However, the number of recalls and false-positive findings were lower for scenario 2 compared with standard combined reading.

Previous studies, based on similar simulated AI-integrated screening setups using different commercial AI solutions to replace one reader with AI also found reduced screen reading volume by up to 50%, with reduced (11,12) or noninferior (13,14) recall rates compared with standard double reading. Findings on cancer detection performance varied between lower (12), noninferior (11,13,14), or higher (11) sensitivity and noninferior (13) or superior (13,14) specificity, depending on the chosen AI threshold or population subset. Another study using the same study population but validating a different AI solution yielded findings consistent with those of the current study (15). However, none of these studies reported or differentiated between readers’ experience and accuracy levels, nor potential differences in screening outcome if the other human reader was to be replaced by AI instead. As shown in this study, use of AI to replace a more experienced reader with an overall better detection accuracy (second reader) rather than a less experienced reader with lower accuracy (first reader) can have significant effect on the outcome of AI-integrated screening (5). Therefore, this distinction may be clinically relevant and should be reported. Furthermore, previous studies either did not include disagreements between the AI and human reader with absent original arbitrations (11) or applied historical second reader decisions (12–14). This results in comparing the standard double reading with an AI-integrated screening with either a more selected sample or a more similar outcome, respectively. This reduces reliability of the reported differences in accuracy between the AI-integrated screening and double reading.

The triage-based setup tested in scenario 3 was investigated by Lauritzen et al (16). The authors reported noninferior sensitivity, higher specificity, and a lower recall rate, with a 62.6% reduction in screen-reading volume. This setup was also evaluated by Frazer et al (17), who found higher sensitivity and specificity and a lower recall rate, with an 80.7% reduction in screen-reading volume. Applying AI as a triage tool as described in scenario 3 could be feasible; however, legal and ethical issues need to be addressed and handled before replacing both radiologists with AI.

It should be noted that the statistically significant differences reported and discussed across the scenarios do not necessarily entail clinically relevant differences. Moreover, AI implementation in double-read mammography screening could alter any predesignated composition of first and second readers based on experience level, as was the case in the current study. Hence, variable reader performance both before and after AI implementation should be considered in workflow adaptations and prior to selecting the appropriate AI-integrated screening setup. Currently, two prospective studies have shown that replacing one reader completely or partially with AI in a real-life double-reading setting can safely reduce workload and maintain CDRs (18,19). Outcome on interval cancers with long-term follow-up is yet to be reported.

Several limitations in this study need to be considered when evaluating the results. The scenarios were all simulations, which imply several assumptions. Thus, the effect of these AI-integrated screening setups in real life, including potential automation bias, is not known. Additionally, the validity of the reference standard could have been affected by different follow-up tests for female individuals who were or were not recalled, the correlation of the radiologist screen-reading decision with the reference standard, and lack of verification for the diagnosis of AI-recalled cases. Finally, the study sample was used for both threshold validation and selection, which can entail overestimation of AI accuracy.

In conclusion, replacing one or both radiologists in double-read mammography screening with AI seems feasible, but there were important differences in accuracy and workload depending on the deployment site in the workflow. A triage-based approach was found to have the best outcome across all performance metrics. However, as it is not possible to replace both readers because of current national guidelines, replacing the first reader with AI represents a feasible solution for implementation. Prospective trials are warranted to validate these scenarios, with particular focus on the effect of AI-integrated screening on clinically relevant outcomes, as well as the impact of replacing either the first or second reader because of their differing experience and accuracy.
